# Efficient Cyberattack Detection Methods in Industrial Control Systems

**DOI:** 10.3390/s24123860

**Published:** 2024-06-14

**Authors:** Piotr Marusak, Robert Nebeluk, Andrzej Wojtulewicz, Krzysztof Cabaj, Patryk Chaber, Maciej Ławryńczuk, Sebastian Plamowski, Krzysztof Zarzycki

**Affiliations:** 1 Institute of Control and Computation Engineering, Faculty of Electronics and Information Technology, Warsaw University of Technology, 00-665 Warsaw, Poland; piotr.marusak@pw.edu.pl (P.M.); robert.nebeluk@pw.edu.pl (R.N.); andrzej.wojtulewicz@pw.edu.pl (A.W.); patryk.chaber@pw.edu.pl (P.C.); sebastian.plamowski@pw.edu.pl (S.P.); krzysztof.zarzycki@pw.edu.pl (K.Z.); 2 Institute of Computer Science, Faculty of Electronics and Information Technology, Warsaw University of Technology, 00-665 Warsaw, Poland; krzysztof.cabaj@pw.edu.pl

**Keywords:** cybersecurity, cyberattack, industrial control system, PID control

## Abstract

The article deals with the issue of detecting cyberattacks on control algorithms running in a real Programmable Logic Controller (PLC) and controlling a real laboratory control plant. The vulnerability of the widely used Proportional–Integral–Derivative (PID) controller is investigated. Four effective, easy-to-implement, and relatively robust methods for detecting attacks on the control signal, output variable, and parameters of the PID controller are researched. The first method verifies whether the value of the control signal sent to the control plant in the previous step is the actual value generated by the controller. The second method relies on detecting sudden, unusual changes in output variables, taking into account the inertial nature of dynamic plants. In the third method, a copy of the controller parameters is used to detect an attack on the controller’s parameters implemented in the PLC. The fourth method uses the golden run in attack detection.

## 1. Introduction

Modern, computerized production systems are equipped with network connectivity, taking advantage of industrial information and communication technologies. This provides the ability to communicate with other facilities and transmit information about the equipment. The interconnection of all systems leads to intelligent factories where production system components and people communicate via networks. In this way, production takes place almost autonomously.

Highly computerized and interconnected facilities open up digital access to equipment, and the benefits of information accessibility come at a price in terms of a higher risk of cyberattack, which poses an increasingly significant threat to industrial networks, e.g., in the energy, robotics, and automotive industries [1,2,3,4,5,6,7,8]. While there are obvious methods to protect against attacks, including the isolation of information technology (IT) and operational technology (OT) networks and the precise definition of network privileges [9], unauthorized access to critical elements of such networks constantly remains a real threat [10].

### 1.1. Related Works

Developing detection mechanisms of possible attacks is, therefore, a key issue. Many data-based detection mechanisms are using artificial intelligence approaches, e.g., support vector data description [11], neural autoencoders [12], k-nearest-neighbors, decision trees, support vector machines, naive Bayes and random forest methods [13], deep neural network models [14,15], genetic or evolutionary algorithms [16], Bayesian networks [17], or machine learning [18]. One can also use classifiers based on statistical properties [19,20,21]. The method described in [22] uses a fusion, adaptive, cubature Kalman filter. The concept presented in [23] relies on convolutional neural networks and Long-Short Term Memory (LSTM) models. A deep-neural-network-based approach is detailed in [24]. Thorough reviews of machine-learning- and artificial-intelligence-based cyberattack detection methods are given in [25,26,27]. On the other hand, simple threshold analysis [28] can also be applied. The relatively simple detection methods do not have the drawbacks of sophisticated data-based detection mechanisms, like complex preparation for application and tailoring to a specific application. Therefore, such relatively simple though effective methods were tested in this work.

### 1.2. Research Gap and Article Contribution

The aforementioned methods based on machine learning and artificial intelligence require precise models. Unfortunately, disturbances and changes in process parameters are inevitable in real-life control systems. As a result, sophisticated, model-based methods are likely to fail in such situations. Therefore, this work is concerned with alternative, model-free methods and one utilizing a golden-run approach, which relies on a non-parametric behavioral model of the entire control system, including the process itself and the control algorithm. The efficiency of all presented methods is evaluated and compared using laboratory software and a hardware environment mimicking industrial control systems. In particular, we confront the model-free methods and the golden-run approach in the case of nonstationary process behavior.

### 1.3. Article Structure

The article is structured as follows. Section 2 presents the laboratory control network, the investigated control plant, and the golden run used in our study. Then, Section 3 discusses efficient cyberattack detection methods and the results obtained when the control system is subjected to attacks; the attack scenarios are described, and the efficiency of the detection methods is investigated. Finally, Section 4 summarizes the article.

## 2. Materials and Methods

**Figure 1:**
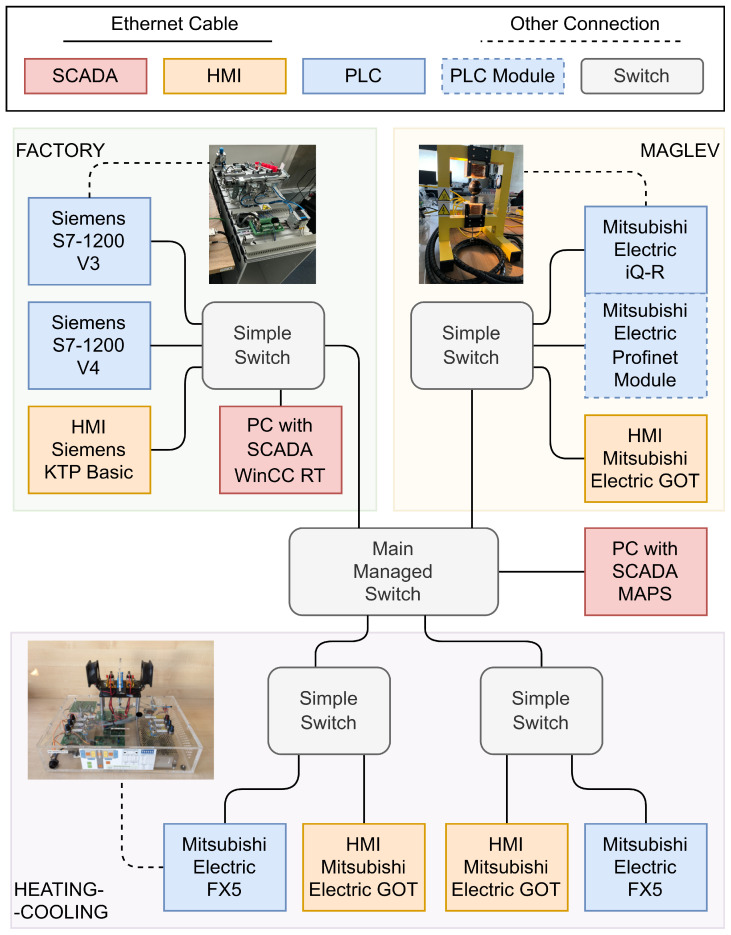
Diagram of the laboratory test bed.

The control plant used in the experiments is part of a more extensive laboratory test bed depicted in Figure 1 used by the authors during testing advanced attack methods [29,30]. Three control processes are available: high-speed magnetic levitation, the MPS FESTO workstation, and the thermal heating and cooling stand. The first and the third processes are continuous, while the second is binary. A typical OT network’s main task is process control. The workstation is enhanced with a local and master computer station with Supervisory Control and Data Acquisition (SCADA) MAPS software ver. 4.0.5.1 to collect and archive data. The central PLC (master) is responsible for managing data from slave controllers and controlling the high-speed magnetic levitation process. The slave controllers perform their local tasks: the FX5U controller, produced by Mitsubishi Electric, Japan, controls the heating and cooling station whereas the S7-1200 controller, produced by Siemens, Germany, controls the FESTO station. Monitoring and diagnostics of the facilities can be performed on local Human–Machine Interface (HMI) panels. Process data are also transferred to the iQ-R master controller. Various protocols have been used, i.e., SLMP, Profinet, Melsoft, Ethernet Simple, Modbus TCP/IP, Modbus RTU, and Siemens S7, marked with appropriate lines on the station diagram. Communication between different devices is achieved using all implemented protocols. Data are exchanged through registers or individual data bits.

### 2.1. Laboratory Test Bed

**Figure 2:**
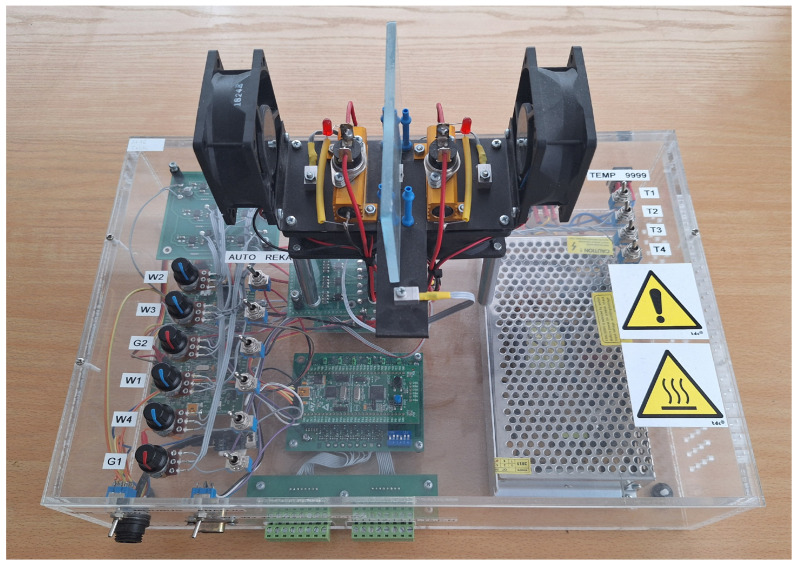
Laboratory thermal control plant.

The experiments were performed in a laboratory heating and cooling thermal control plant, where the user influences the temperature distribution through controllable fans and heaters. The bench can be controlled manually or via an automation system using the Modbus communication protocol. A picture of the laboratory thermal stand is presented in Figure 2.

The control plant has six manipulated variable (MV) inputs:

- FLU, FLB, FRU, and FRB fans, with values from 0 to 100;
- HL and HR heaters, with values from 0 to 100.

And seven process variable (PV) outputs:

- TL, TM, TR, and TF bench temperature, with values from −55.0 °C to +125.0 °C;
- TA ambient temperature, with values from −55.0 °C to +125.0 °C;
- C current measurement;
- V voltage measurement.

A Pulse-Width Modulation (PWM) signal controls the actuators. The temperature sensors communicate internally using the OneWire bus, while the current and voltage measurements are carried out using dedicated electronics. All input and output signals are available via the Modbus protocol.

If the control of the fans is constant, then the control plant is linear, i.e., the temperatures linearly depend on the heater control signals. Changes in fan load cause changes in the dynamics and statics (amplification) of the control plant. The load of the left fan was set to $z_{\mathrm{FLU}}=50\%$ during the research. It should be noted that the thermal process under consideration is slow, with temperatures settling after about 150 s.

The control plant is versatile and allows different controllers to be tested in different configurations of control systems. For current research, the number of signals used is limited to the following:

- $u_{\mathrm{HL}}$ is the left heater control signal (manipulated variable in the tested control system); $u_{\mathrm{HL}}^{\min}=0\%$, $u_{\mathrm{HL}}^{\max}=100\%$;
- $y_{\mathrm{TL}}$ is the temperature measured on the left-hand-side of the laboratory stand (process variable in the tested control system); $y_{\mathrm{TL}}^{\min}=20$°C, $y_{\mathrm{TL}}^{\max}=90$°C;
- $z_{\mathrm{FLU}}$ is the left fan control signal (disturbance variable in the tested control system); $z_{\mathrm{FLU}}^{\min}=0\%$, $z_{\mathrm{FLU}}^{\max}=100\%$.

The PID control algorithm was implemented on a PLC. The controlled variable is $y_{\mathrm{TL}}$, and the manipulated variable is $u_{\mathrm{HL}}$. The PID control algorithm derives the value of the control signal $u_{\mathrm{HL}}$ using the measurement of the output variable $y_{\mathrm{TL}}$. Communication with the laboratory control plant is performed using the Modbus RTU protocol.

### 2.2. Control System Operation in Nominal Conditions

**Figure 3:**
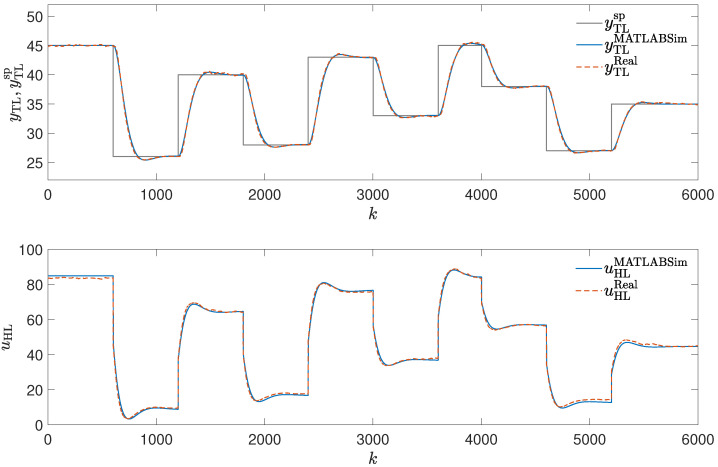
Responses of the control system operating in nominal conditions.

The responses obtained in the tested control system are depicted in Figure 3. The PID controller was tuned in such a way that the control system was sufficiently fast, with a relatively short settling time and reasonable rise time; the overshoot was very small; and the control signal changed smoothly. Thus, the obtained control quality was good. The temperature plot is taken as a reference plot (golden run) used for comparisons in the implementation of Method #4.

### 2.3. Attack Scenarios and Detection Methods

The tested detection methods are designed in such a way that they:

- Are easy to implement;
- Are efficient in detecting a cyberattack;
- Generate few false alarms.

All evaluations were carried out using the laboratory test bed described in Section 2.1, which mimics real industrial control systems, i.e., it relies on software and hardware solutions used in industrial practice.

#### 2.3.1. Method #1: Verification of the Control Value

##### Attack Scenario

**Figure 4:**
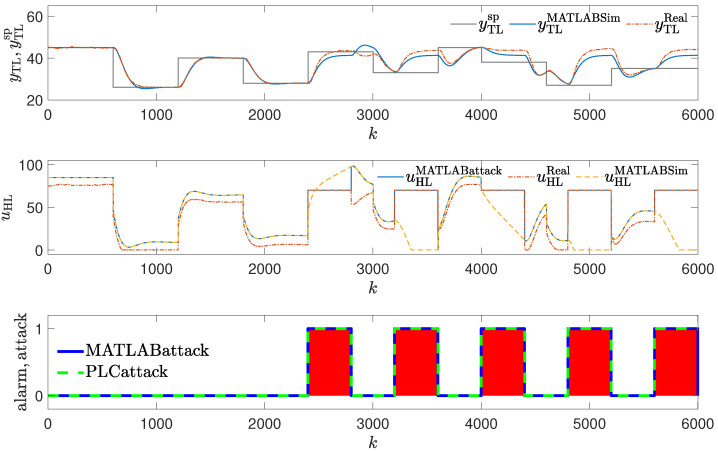
Responses of the control system operating during the first attack; detection method: verification of the control value with its copy (red blocks signal attack detection).

In the first attack scenario, the control signal is frozen on the value of $u_{\mathrm{hl}}=70\;\%$ for $T_{\mathrm{atack}}=400$ s, then the controller is allowed to operate for $T_{\mathrm{noatack}}=400$ s, then the attack is repeated, and so on. The attack repeats five times. The periods during which the attack is performed are marked with blue in Figure 4. During the attack, the value of the output variable tends to settle around 44. The controller tries to achieve the setpoint value every time the attack stops. It is able to achieve its goal, but every time the setpoint is reached, a new attack is performed.

##### Detection Mechanism

The first method under investigation concentrates on the analysis of the control signal. It verifies whether the value sent to the control plant in the previous step is the actual value generated by the controller. If the verification result is negative, then an alarm is reported.

#### 2.3.2. Method #2: Detection of Sudden Change in Output Variable

##### Attack Scenario

**Figure 5:**
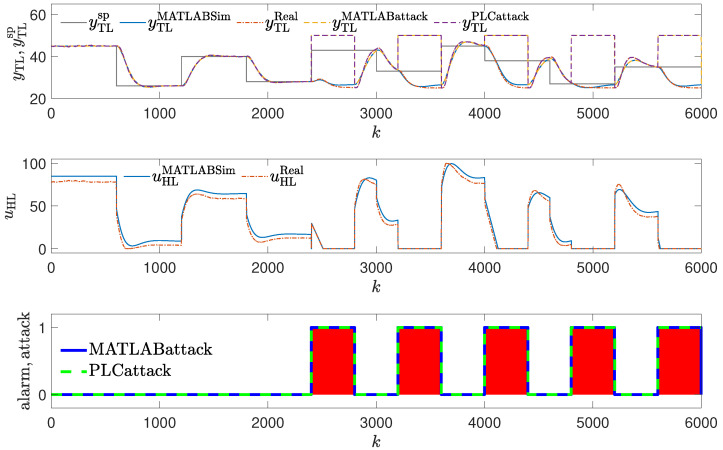
Responses of the control system operating during the second attack; detection of a sudden change in output variable using the copy of the temperature measurement (red blocks signal attack detection).

In the second attack scenario, the process variable (temperature) is passed to the controller as the measurement is frozen on the value of $y_{\mathrm{tl}}=50\;C$ for $T_{\mathrm{atack}}=400$ s, then the proper value of temperature is allowed to be sent to the controller for $T_{\mathrm{noatack}}=400$ s, then the attack is repeated, and so on. The attack repeats five times. The value sent to the controller as the temperature measurement is indicated with the dashed, red line in the upper subplot of Figure 5, and the real value is marked with the solid, blue line. The faked process variable value is larger during the attack than the setpoint. Therefore, the control signal tends to settle on 0 % (achieves the minimal possible value), and the real process variable tends toward a small value below the setpoint (it is the value close to the ambient temperature). Once the attack is stopped, the controller tends to achieve the setpoint value, but a new attack causes the process variable to get away from the setpoint.

##### Detection Mechanism

In each sampling instant, the measured temperature value is compared with the value obtained by the controller. For the mechanism to work, there must be two channels measuring the temperature created on the PLC in order to implement this security measure. Thus, temperature values exist in two different registers, but only one is used by the controller, and the attacker also overwrites this copy.

#### 2.3.3. Method #3: Copy of Controller Parameters Used to Detect an Attack

##### Attack Scenario

**Figure 6:**
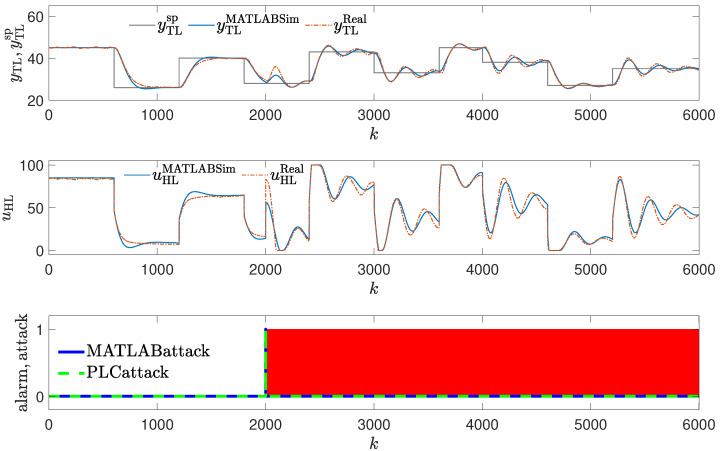
Responses of the control system operating during the third attack (significant change in the PID controller parameters); copy of controller parameters used to detect an attack (red blocks signal attack detection).

Two attack scenarios were tested. In the first one, it is assumed that the attacker changes the parameters of the Proportional–Integral (PI) controller in such a way that control quality deteriorates visibly (see Figure 6). It is assumed that K=0.3, $T_{I}=20$ (the nominal values of the parameter are K=0.2, $T_{I}=50$). The oscillations in the obtained responses occur. Interestingly, as shown in Table 1, the value of Mean Squared Error (MSE) drops compared to the golden run (but the Mean Absolute Error (MAE) increases); this illustrates that sole analysis of the values of control quality indexes may be insufficient to detect the attack.

**Figure 7:**
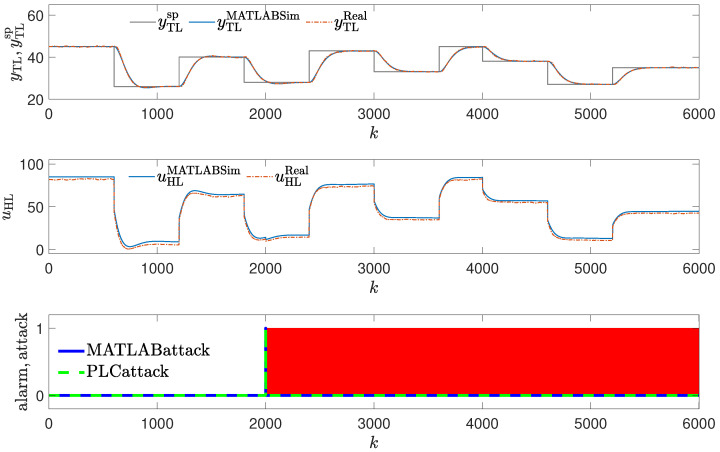
Responses of the control system operating during the third attack (small change in the PID controller parameters); copy of controller parameters used to detect an attack (red blocks signal attack detection).

In the second attack scenario, it is assumed that the parameters are altered slightly so that the operation of the control system deteriorates in such a way that it is difficult to detect the attack just by visual analysis of the control system response by the operator (see Figure 7). A minor change in the controller parameters was made (K=0.2, $T_{I}=60$); thus, only the integration time was slightly changed. The obtained responses are (visually) very close to the golden run; MSE and MAE deteriorate only slightly.

##### Detection Mechanism

In each sampling instant, the values of the controller parameters are compared with the values of their copy (gain *K* and integration time $T_{I}$). For the mechanism to work, there must be a copy of the controller parameters located in a safe place, outside reach of the attacker, ideally, in a place the attacker does not know about or in such a place so that the attacker cannot alter the values of the parameter copies.

#### 2.3.4. Method #4: Using Golden Run in an Attack Detection

##### Attack Scenario

**Figure 8:**
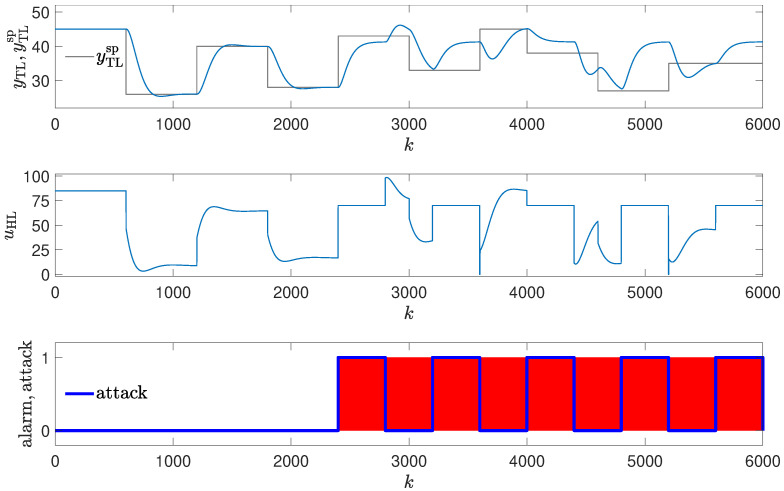
Responses of the control system operating during the first attack; detection method: golden run; simulation experiments (red blocks signal attack detection).

**Figure 9:**
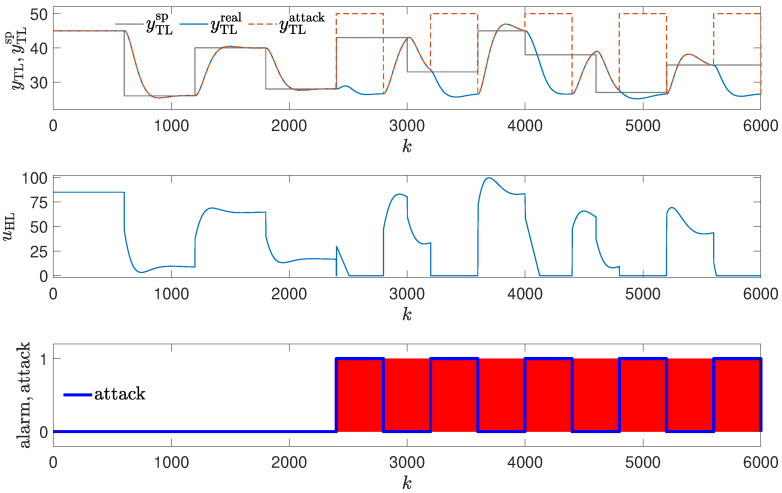
Responses of the control system operating during the second attack; detection method: golden run; simulation experiments (red blocks signal attack detection).

**Figure 10:**
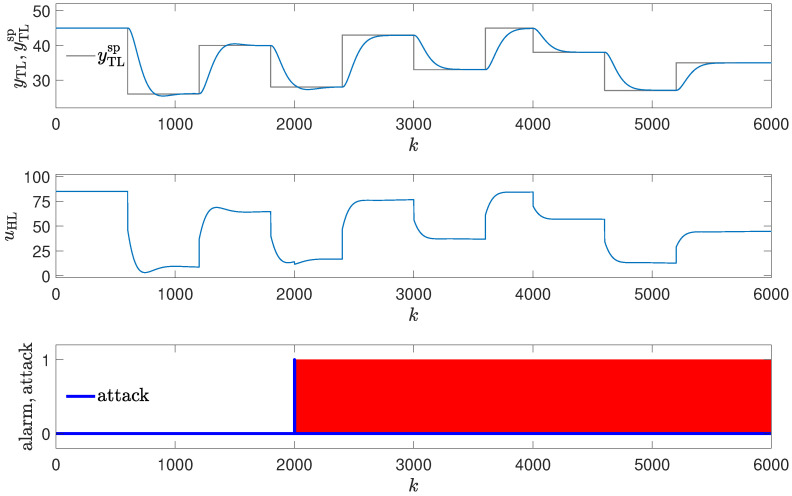
Responses of the control system operating during the third attack (small change in PID parameters); detection method: golden run; simulation experiments (red blocks signal attack detection).

**Figure 11:**
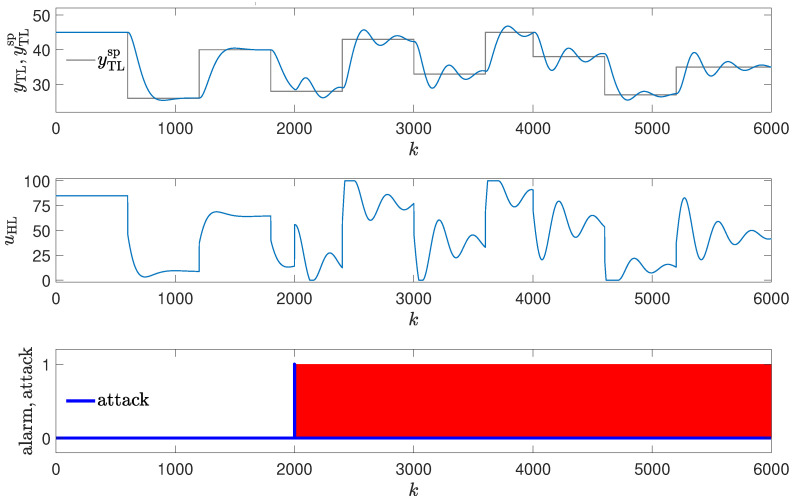
Responses of the control system operating during the third attack (large change in PID parameters); detection method: golden run; simulation experiments (red blocks signal attack detection).

**Figure 12:**
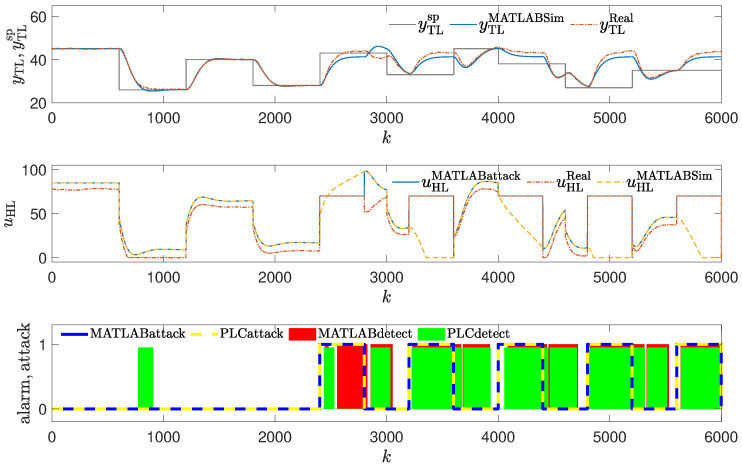
Responses of the control system operating during the first attack; detection method: golden run; laboratory experiments.

**Figure 13:**
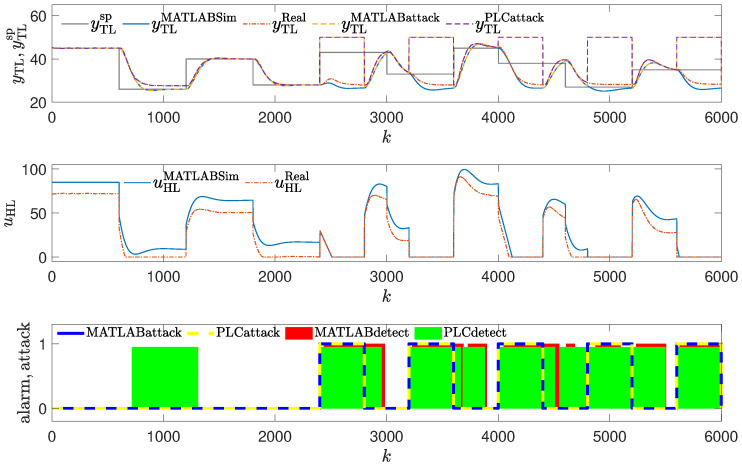
Responses of the control system operating during the second attack; detection method: golden run; laboratory experiments.

**Figure 14:**
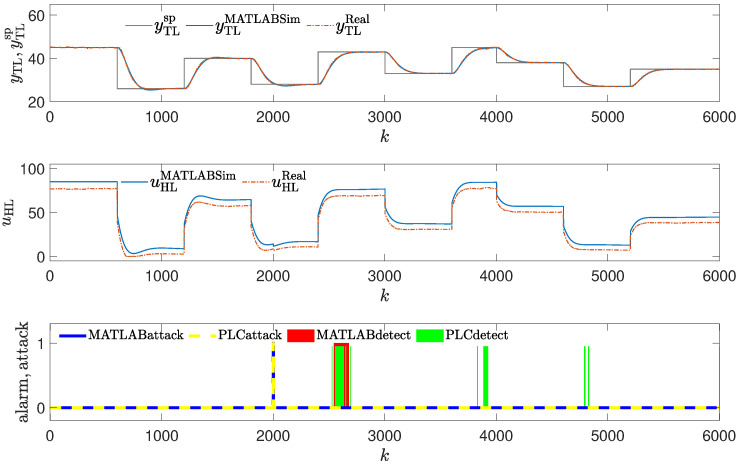
Responses of the control system operating during the third attack (small change in PID parameters); detection method: golden run; laboratory experiments.

**Figure 15:**
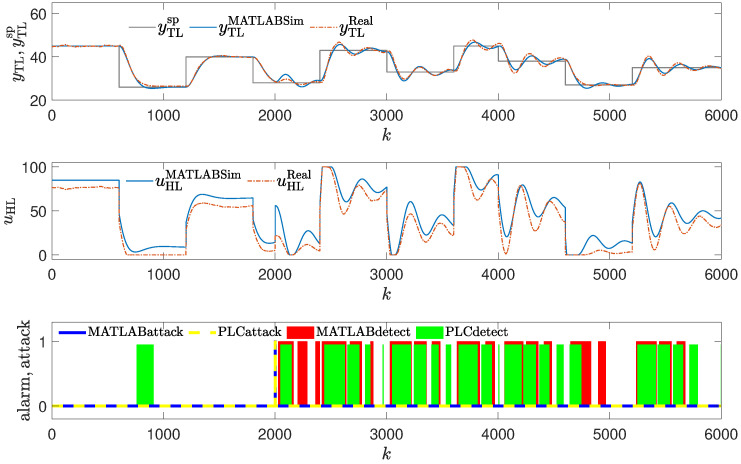
Responses of the control system operating during the third attack (large change in PID parameters); detection method: golden run; laboratory experiments.

The method was tested using the same attack scenarios as already described:

- To conduct the experiments depicted in Figures 8 and 12,the attack scenario described in Section 2.3.1, also used to obtain the result shown in Figure 4, was applied.
- To conduct the experiments depicted in Figures 9 and 13, the attack scenario described in Section 2.3.2, also used to obtain the result shown in Figure 5, was applied.
- To conduct the experiments depicted in Figures 10, 11, 14 and 15, the attack scenarios described in Section 2.3.3, also used to obtain the result shown in Figures 6 and 7, were applied.

##### Detection Mechanism

The mechanism uses the golden run (in our case, the responses depicted in Figure 3). Then, the absolute value of the difference between the current temperature and the temperature from the reference response ($|T_{\mathrm{real}}-T_{\mathrm{gr}}|$) is calculated at each time step. If the difference exceeds the assumed limit, an alarm is reported. In the case of the experiments shown in Figures 8–11, the limit equal to 0 was assumed; in the case of the experiments shown in Figures 12–15, the limit equal to 1 was assumed. However, the tests in the simulation environment were also performed for other values of this parameter, and the mechanism gave the same result (in terms of attack detection) up to the limit equal to 6.

## 3. Results and Discussion

In the current section, the effectiveness of all discussed detection methods is studied. The first three model-free methods require the availability of process measurements, while the golden-run approach relies on the availability of the behavioral controlled process model.

### 3.1. Method #1: Verification of the Control Value

The detection mechanism was used to detect the attack and invoke the alarm. Thus, every time the last value generated by the controller is not the value sent to the control plant, an alarm is reported (red blocks in Figure 4). The efficiency of the method is excellent. Every time the attack occurs, it is detected at once and in each instant it is present. The method can fail only when the attacker generates the same control value as the controller. However, in such a case, the operation of the control system would not have deteriorated as a result of the attack.

### 3.2. Method #2: Detection of Sudden Change in Output Variable

The detection mechanism used to detect the attack invoked the alarm every time temperature values from two registers differ (red blocks in Figure 5). The method’s efficiency is excellent because every time the attack occurs, it is detected at once. The method can fail when the attacker generates a temperature value equal to the actual temperature. Still, the operation of the control system would not have deteriorated as a result of the attack. The other possibility is that the attacker alternates values in both registers, but they must know the detection mechanism and location of the measurement copy.

### 3.3. Method #3: Copy of Controller Parameters Used to Detect an Attack

The detection mechanism detects the attack every time values of the controller parameters differ from their copy (red blocks in Figure 6 and Figure 7), then the alarm is invoked. The effectiveness of this method is excellent, as the attack is detected as soon as it is launched. It does not matter whether the change in parameters is small or significant. The method is, therefore, more difficult to cheat than the operator. The method can fail if the attacker alternates values of the parameters in the copy stored for reference. Still, they must know about the detection mechanism and the location of the copy of the controller parameters.

### 3.4. Method #4: Using Golden Run in an Attack Detection

The detection mechanism detects the attack every time values of the reference response differ enough from the responses obtained from the real control plant (red blocks in Figure 8, Figure 9, Figure 10 and Figure 11), then the alarm is signaled. The method detects the beginning of the attack reliably, but unlike methods #1 and #2, it signals the attack constantly, even if currently the attack is not performed (because the registered golden run differs from the responses generated in the real control system).

#### Obtained Results: Laboratory Experiments

The experiments in the simulation environment suggested that the method, though having some drawbacks, should work well. However, during the laboratory tests, very interesting results were obtained. Due to the nonstationary characteristic of the process, the method had problems detecting the attacks (it generated false alarms in situations depicted in Figure 12, Figure 13, Figure 14 and Figure 15, and was late in detecting the attack in the situation depicted in Figure 14).

The drawbacks of model-based methods (though a simplified model, in the form of a waveform, is used) have made their presence known. If the process is nonstationary, its model must be updated if a significant change occurs. It means that if one wants to be sure the proper model is employed, it must be obtained (or at least updated) each time the method is used, and in some cases, it is unacceptable. In all cases, when this is necessary, it causes significant inconvenience.

### 3.5. Comparison of Performance Metrics

Table 2 compares performance metrics obtained for the laboratory stand for a given attack scenario and the detection method. It is clear that the detection rate of model-free methods is 100% whereas the false positive rate does not exceed 0.1%. Similarly, precision is not less than 99.8%. The golden-run-based method gives poor performance metrics, e.g., the precision rate for the first and the second attacks is approximately 55%. In the case of the third attack, the detection rate is very poor, especially when a small change in the controller parameters is made; the detection rate amounts to only 3.55%.

Finally, we evaluated the discussed simulation methods. Table 3 compares performance metrics obtained in the simulation experiment for a given attack scenario and the detection method. Even in the simulated environment, the performance of the golden-run-based method is worse than that of model-free methods working in the laboratory environment.

## 4. Conclusions

Three effective, easy-to-implement, and relatively robust methods for detecting attacks on the control signal, output variable, and the PID controller parameters have been implemented and tested; also, a method using a simplified model—a waveform recorded during the golden run—has been considered. The first three methods, namely, Method #1: verification of the control signal value sent to the control plant, Method #2: detection of sudden changes in output variables, and Method #3: using a copy of the controller parameters to detect an attack, can be applied online and are very effective. They detect an attack almost at once as soon as it is performed and are relatively hard to fool.

The Method #4 uses the golden run in attack detection. A feature of this method is that under favorable circumstances (for stationary processes), it detects the occurrence of the attack fast. Still, once the attack is detected, the method signals it constantly. Therefore, in the case of attack scenarios from Section 2.3.1 and Section 2.3.2 it invokes the alarm even if the attack is halted for a while. Unfortunately, in the case of a nonstationary process, its significant drawbacks manifested, showing the need for relatively frequent model updating. On the other hand, the method can also detect unusual behavior of the control system subjected to attack scenarios that have not been researched in the article.

In the future, we plan to design more sophisticated detection methods for nonstationary control plants, i.e., with time-varying parameters. Secondly, as a result of this work, we have found that model-based detection methods are very susceptible to the control plant’s parameter changes. Hence, this complex problem is hard to solve and very important in the case of nonstationary processes.

## Figures and Tables

**Table 1:** Comparison of control quality indicators obtained for the laboratory stand for a given attack scenario and the detection method.

Attack Scenario	Detection Method	MSE	MAE
No attack	–	$1.7362\times 10^{1}$	$1.9370\times 10^{0}$
First attack	MV copy	$4.0905\times 10^{1}$	$4.2441\times 10^{0}$
First attack	Golden run	$3.8889\times 10^{1}$	$4.1876\times 10^{0}$
Second attack	PV copy	$5.3678\times 10^{1}$	$4.8352\times 10^{0}$
Second attack	Golden run	$3.9102\times 10^{1}$	$4.1863\times 10^{0}$
Third attack v. 1	Copy of parameters	$1.8223\times 10^{1}$	$2.0957\times 10^{0}$
Third attack v. 1	Golden run	$1.7591\times 10^{1}$	$2.0220\times 10^{0}$
Third attack v. 2	Copy of parameters	$1.7877\times 10^{1}$	$2.4675\times 10^{0}$
Third attack v. 2	Golden run	$1.6701\times 10^{1}$	$2.2712\times 10^{0}$

**Table 2:** Comparison of performance metrics obtained for the laboratory stand for a given attack scenario and the detection method.

Attack Scenario	Detection Method	Detection Rate	False Positive Rate	Precision	Recall	Time to Detect (First Attack/Mean)
First attack	MV copy	100.0%	0.05%	99.9%	100.0%	0.0/0.0
First attack	Golden run	077.5%	30.2%	56.2%	077.5%	38.0/37.2
Second attack	PV copy	100.0%	0.1%	99.8%	100.0%	0.0/0.0
Second attack	Golden run	100.0%	41.0%	54.95%	100.0%	0.0/0.0
Third attack v. 1	Copy of parameters	100.0%	0.0%	100.0%	100.0%	0.0/n.a.
Third attack v. 1	Golden run	3.55%	0.0%	100.0%	3.55%	527.0/n.a.
Third attack v. 2	Copy of parameters	100.0%	0.0%	100.0%	100.0%	0.0/n.a.
Third attack v. 2	Golden run	51.3%	5.5%	94.91%	51.3%	48.0/n.a.

**Table 3:** Comparison of performance metrics obtained in the simulation experiment for a given attack scenario and the detection method.

Attack Scenario	Detection Method	Detection Rate	False Positive Rate	Precision	Recall	Time to Detect (First Attack/Mean)
First attack	Golden run	84.8%	28.5%	59.8%	84.8%	49.0/45.2
Second attack	Golden run	83.8%	23.38%	64.19%	83.8%	38.0/44.0
Third attack v. 1	Golden run	3.28%	0.0%	100.0%	3.28%	545.0/n.a.
Third attack v. 2	Golden run	55.85%	0.0%	100.0%	55.85%	29.0/n.a.

## Data Availability

Data is contained within the article.

## Abbreviations

HMI	Human–Machine Interface
IT	Information technology
MAE	Mean Absolute Error
MSE	Mean Squared Error
MV	Manipulated variable
OT	Operational technology
PI	Proportional–Integral
PID	Proportional–Integral–Derivative
PLC	Programmable Logic Controller
PV	Process variable
PWM	Pulse-Width Modulation
SCADA	Supervisory Control and Data Acquisition
